# Fine-tuning improves deep learning-based digital phenotyping of lettuce genetic resources

**DOI:** 10.3389/fpls.2026.1970840

**Published:** 2026-10-09

**Authors:** Eunju Lee, Yuseok Jeong, Hyo Jun Bae, Yoon-Jung Lee, Seong-Hoon Kim, Young-Wang Na, Hwan-suk Cheong, Inchan Choi

**Affiliations:** 1 Department of Agricultural Engineering, National Institute of Agricultural Sciences, Rural Development Administration, Jeonju, Republic of Korea; 2 National Agrobiodiversity Center, National Institute of Agricultural Sciences, Rural Development Administration, Jeonju, Republic of Korea; 3 Agricultural AI and Data Division, Rural Development Administration, Jeonju, Republic of Korea

**Keywords:** agricultural genetic resources, deep learning, digital phenotyping, genebank, *Lactuca sativa*

## Abstract

**Introduction:**

Morphological characterization of agricultural genetic resources relies largely on visual assessment by experts, and applying the same criteria repeatedly to large collections is labor-intensive. Image-based deep learning can support this process, but systematic evidence on training strategies suited to genebank trait data remains limited.

**Methods:**

This study targeted six morphological traits of lettuce genetic resources characterized according to the test guidelines of the International Union for the Protection of New Varieties of Plants (UPOV). Using genebank images accumulated from 2012 to 2025, six convolutional neural networks were trained with three training strategies under identical conditions, and their performance was compared.

**Results:**

Fine-tuning achieved the highest mean macro F1-score in 35 of the 36 backbone–trait combinations and significantly outperformed training from scratch and feature extraction, whereas no single backbone was superior across all traits. Misclassifications were concentrated in morphologically adjacent classes rather than randomly distributed, and for the ordinal traits, errors spanning two or more class levels were rare.

**Discussion:**

These patterns suggest that the remaining classification difficulty was associated in part with the morphological continuity between neighboring classes. The attainable performance also differed among traits, distinguishing traits for which the training strategy alone achieved relatively high performance from those for which it did not. These results provide empirical evidence for selecting training strategies and for determining the role of model-assisted characterization on a trait-by-trait basis in genebanks.

## 1 Introduction

Agricultural genetic resources are essential biological resources for future food security and sustainable agriculture, serving as a source of the genes required for the development of new cultivars and for responses to climate change (Aubry, 2023; Ghamkhar et al., 2025). However, the value of genetic resources does not end with their conservation alone. A core mission of genebanks worldwide is to systematically characterize the morphological and agronomic attributes held by each accession and to compile them into databases so that researchers and breeders can make use of them (Weise et al., 2020; Volk et al., 2021). Genebanks in countries around the world therefore continuously characterize the morphological characteristics and agronomic traits of their genetic resources and build these into databases, which serve as reference data for the conservation, management, and utilization of genetic resources (Halewood et al., 2018).

Such trait characterization is performed according to internationally standardized criteria (Bazakos et al., 2024; Huh et al., 2024; Alemu et al., 2025). Representative examples are the Guidelines for the Conduct of Tests for Distinctness, Uniformity and Stability issued by the International Union for the Protection of New Varieties of Plants (UPOV) and the crop-specific descriptors, both of which are commonly used as characterization criteria by major seed banks and genetic resource institutions worldwide (Lootens et al., 2013; Kadoglidou et al., 2023). Such standardized trait information serves not only for cultivar identification but also as indispensable baseline data for testing the redundancy of genetic resources, evaluating their characteristics, searching for breeding materials, and utilizing genetic resources.

At present, however, most trait characterization still depends on visual evaluation by experts. The results of this approach can vary with the experience and proficiency of the evaluator, and assessments of the same trait can differ between evaluators. In addition, genebanks must repeatedly characterize thousands to tens of thousands of accessions each year according to the same criteria, which demands considerable time and labor (Conejo-Rodríguez et al., 2024). Image-based digital phenotyping can therefore serve as a tool to support repetitive expert assessment and facilitate more consistent application of predefined characterization criteria, although its ability to reduce inter-observer variability or reproduce expert assessment requires separate validation.

Recent advances in computer vision and deep learning have led to the rapid adoption of digital phenotyping in agriculture (Li et al., 2020; Sakeef et al., 2023; Wang et al., 2026). Its potential has also been recognized in genebanks, where phenomics and image-based analysis are being introduced to complement conventional characterization (Kienbaum et al., 2021; Volk et al., 2021; Kim et al., 2023; Ghamkhar et al., 2025). Whereas general image classification studies focus on raising classification accuracy, the ultimate goal of digital characterization in genebanks is the continuous production of objective and reproducible trait information based on international standards.

Actual agricultural genetic resource data, however, have characteristics that differ from those of general computer vision data. Images of genetic resources may be acquired under heterogeneous imaging conditions, so illumination and background are not necessarily uniform, and the scale of the data is limited by the difficulty of securing accessions (Le Floch et al., 2026). In genebanks, images may also be accumulated over long periods using different equipment and acquisition settings. Class distributions within a trait are also frequently imbalanced, and class imbalance has been shown to have a detrimental effect on the classification performance of CNNs (Buda et al., 2018). In addition, some morphological trait-classification tasks can be regarded as fine-grained classification problems because neighboring categories may differ only subtly, a challenge also reported in plant phenotype classification (Yuan et al., 2022). For traits expressed as increasing levels of intensity, the classes are also ordered, so a disagreement between adjacent categories differs in severity from one spanning several levels (Cohen, 1968). In a practical genebank setting, therefore, identifying a training strategy that performs consistently under limited and imbalanced data is as important as selecting a high-performing network architecture.

Lettuce (*Lactuca sativa* L.) was selected as the subject of this study. Lettuce is a globally important leafy vegetable and a representative genetic resource crop for which diverse accessions are conserved. Morphological traits such as leaf shape, leaf blistering, leaf margin serration, plant type, head type, and heading degree are diverse in their expression and are used as major distinguishing traits in the UPOV test guidelines, and in genetic resources, the presence and degree of their expression can differ among individual plants. These traits also differ in the number and frequency distribution of classes as well as in the degree of morphological separation between categories, making lettuce suitable for evaluating image-based phenotypic classification across traits with different class structures and degrees of morphological complexity.

A wide range of studies using deep learning-based techniques has recently been conducted (Taghavi Namin et al., 2018; Altuntaş et al., 2019; Zhang et al., 2022; Van et al., 2025). The performance of deep learning models is affected by the amount and distribution of the training data, and more stable results are obtained when sufficient labeled data have been collected. In practical research settings, however, building such data often requires considerable cost and time (Zhuang et al., 2020). In agriculture, large labeled datasets are particularly scarce because data collection is time-consuming and labor-intensive and annotation requires expert knowledge. Transfer learning has therefore been widely adopted to adapt pretrained models to agricultural tasks, including fine-grained plant phenotype classification (Yuan et al., 2022; Hossen et al., 2025).

Training from scratch, feature extraction, and fine-tuning are representative strategies for training CNNs on limited data, differing in whether and how ImageNet-pretrained weights are used. Previous studies have established the usefulness of transfer learning for agricultural image analysis and have compared transfer learning strategies on plant classification benchmarks (Kaya et al., 2019; Hossen et al., 2025). These findings, however, do not directly establish which training strategy is most appropriate for heterogeneous genebank trait data. In long-term genebank image datasets, such data characteristics can co-occur, including heterogeneous images, imbalanced class distributions, and subtle or ordered differences between categories in some traits. Systematic empirical evidence comparing major CNN training strategies under matched conditions across multiple backbones and multiple morphological traits using such archival genebank image data remains limited. Consequently, it remains unclear whether the relative advantage of a training strategy is consistent across traits, or whether the remaining classification limitations are more strongly associated with the training strategy or with trait-specific morphological and data characteristics.

Therefore, to provide a systematic evaluation, this study compared six representative convolutional neural networks and three training strategies under identical conditions using images of lettuce genetic resource traits compiled by the National Agrobiodiversity Center, National Institute of Agricultural Sciences, Rural Development Administration. Repeated experiments were used to assess run-to-run variability and the statistical significance of performance differences among training strategies. In addition to conventional classification metrics, misclassification patterns and class-distance-based metrics for ordinal traits were examined to interpret trait-dependent differences and limitations in classification performance.

Accordingly, this study addressed three research questions: (1) Do training from scratch, feature extraction, and fine-tuning differ significantly in classification performance for genebank trait images? (2) Is the relative effect of training strategy consistent across CNN backbones and traits with different class structures and morphological characteristics? (3) When classification performance is limited, are errors randomly distributed or concentrated between morphologically adjacent classes? Through these analyses, this study aims to provide empirical evidence for selecting and interpreting deep learning training strategies for digital phenotyping in practical genebank applications.

## 2 Materials and methods

The overall pipeline of this study is shown in **Figure 1**. In the data collection stage, lettuce plants are grown and photographed, after which experts label them by trait, thereby building up a genetic resource database. Images of the target traits are retrieved from the accumulated database and subjected to preprocessing, which comprises separation into individual specimens, size normalization, and splitting of the dataset for training. Convolutional neural network backbones and training strategies are then crossed, and each combination is trained under identical conditions. Finally, the trained models are evaluated using various metrics and analyzed through confusion matrices and Grad-CAM. The results obtained in this way can be extended in various ways to the digital phenotyping framework of a genebank. The details of each stage are described in the following sections.

**Figure 1:**
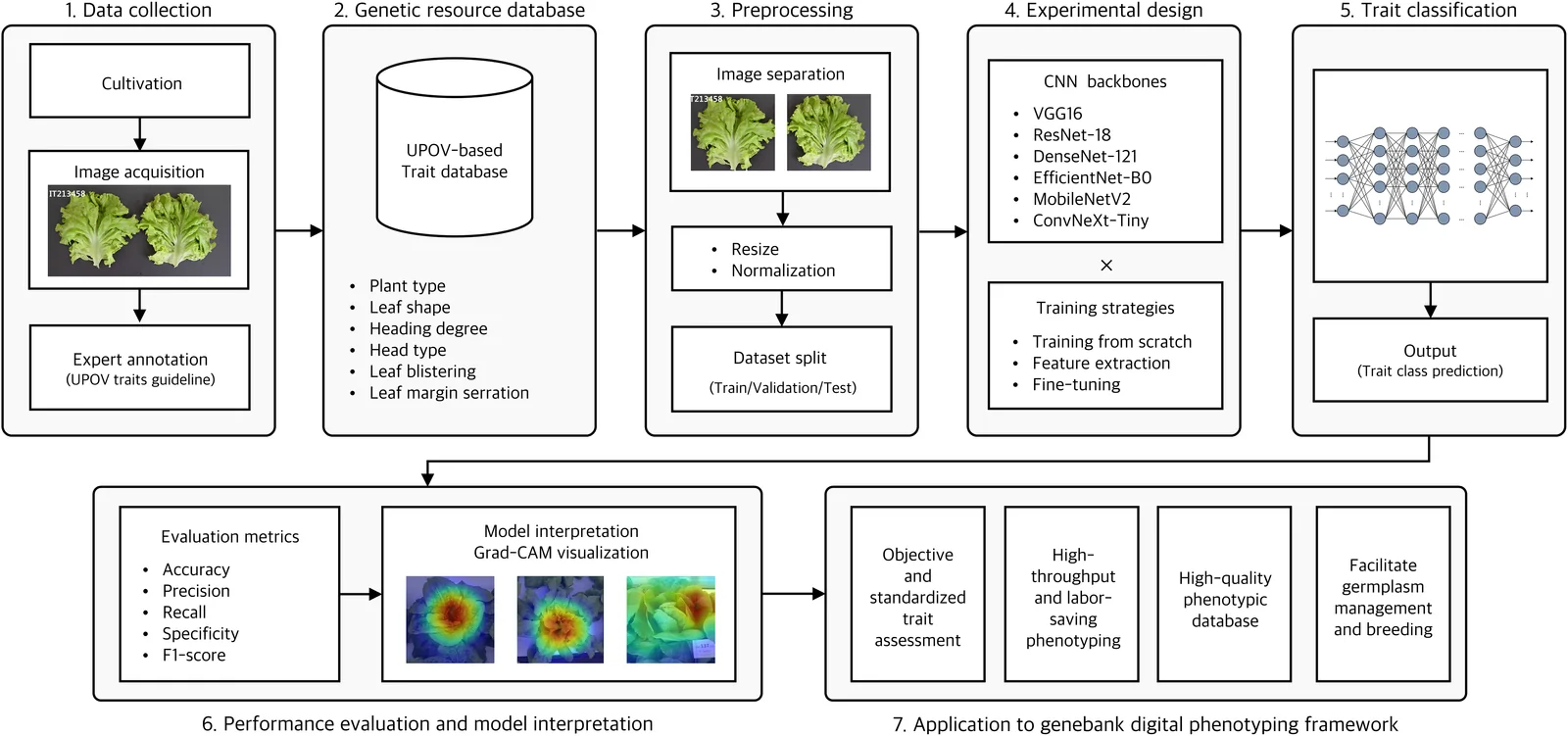
Overall research pipeline for the classification of lettuce genetic resource traits.

### 2.1 Dataset

The lettuce plant and leaf datasets used in this study are publicly available through the RDA Genebank. These data were collected in accordance with the characterization criteria of the National Agrobiodiversity Center, which are based on the UPOV test guidelines.

The image acquisition conditions varied with the period of photography. The cameras used included a Canon 800D (18 mm), a Samsung WB1000, and a Samsung SM-G960N, and the imaging distance ranged from approximately 1.5 m outdoors to approximately 3~50 cm indoors. White or black mulching film was used as the background, and the lighting conditions were a mixture of outdoor natural light and indoor artificial light. No separate color correction was performed, and photography was carried out on multiple days over several years.

The datasets comprise six traits in total. Leaf shape, leaf blistering, and leaf margin serration were evaluated at the leaf level, whereas plant type, head type, and heading degree were assessed based on whole-plant morphology. The datasets were constructed from characterization data and images collected from 2012 to 2025. For each evaluation, at least 10 plants per accession were cultivated, and trait characterization was conducted at the optimal growth stage. Representative leaves expressing the respective phenotypes were collected from individual plants and photographed separately for the leaf-level traits, whereas images of the whole plant were acquired for the whole-plant traits.

The original images contained either a single specimen or multiple specimens within one frame. Because this study used a classification model that assigns a single class to each input, preprocessing was performed to separate each specimen from the original image for specimen-level training (**Figure 2**). Since the traits in this study use morphological information itself, such as the outline and proportions of the leaf or plant, as the criterion for discrimination, trait information can be lost if the aspect ratio is distorted during resizing. To prevent this, all images were standardized to a size of 224×224×3 by downscaling them while preserving the original aspect ratio and filling the remaining area with zeros.

**Figure 2:**
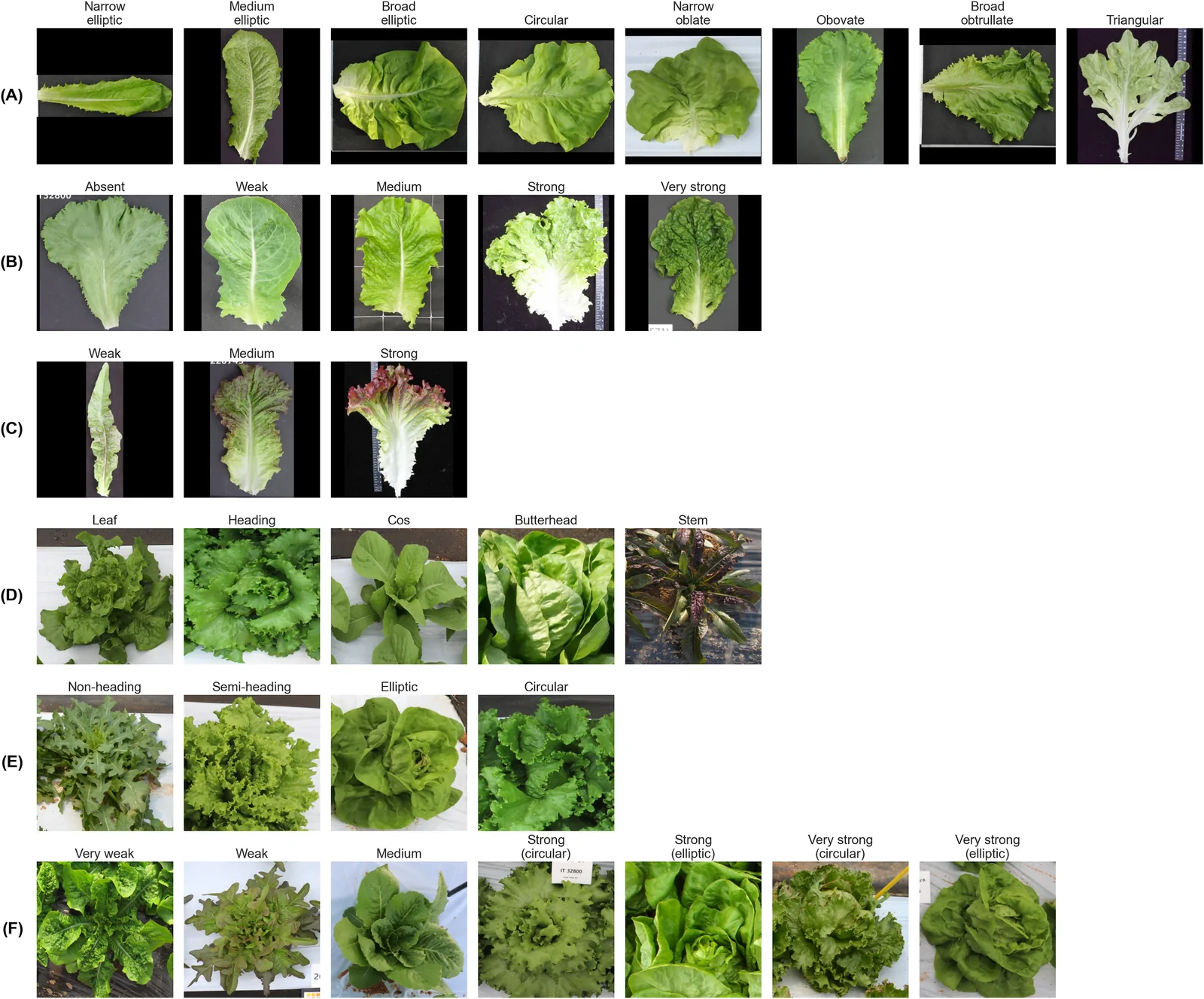
Examples of the preprocessed dataset by trait and class. **(A)** Leaf shape. **(B)** Leaf blistering. **(C)** Leaf margin serration. **(D)** Plant type. **(E)** Head type. **(F)** Heading degree. The label above each image denotes the class.

Because phenotypic expression could vary among individual plants within the same accession, the phenotype observed in each individual leaf or plant, rather than accession identity, was used as the unit of classification. The samples for each trait were randomly divided at the individual-sample level, without grouping by accession, into training, validation, and test sets at a ratio of 6:2:2. The models were trained on the training data, the trend in performance during training was monitored on the validation data, and the test data were used only for the final performance evaluation and not for model training or selection.

The number of classes and the size of the six datasets are given in **Table 1**, and the detailed class distribution of each trait by form of expression is presented as a bar chart so that the degree of imbalance in each trait can be seen (**Figure 3**). The number of samples per class in each dataset is presented in **Supplementary Table S1**.

**Table 1:** Number of classes and dataset size for each trait.

Trait	Number of classes	Total images
Leaf shape	8	1,140
Leaf blistering	5	1,202
Leaf margin serration	3	912
Plant type	5	6,510
Head type	4	6,877
Heading degree	7	6,982

**Figure 3:**
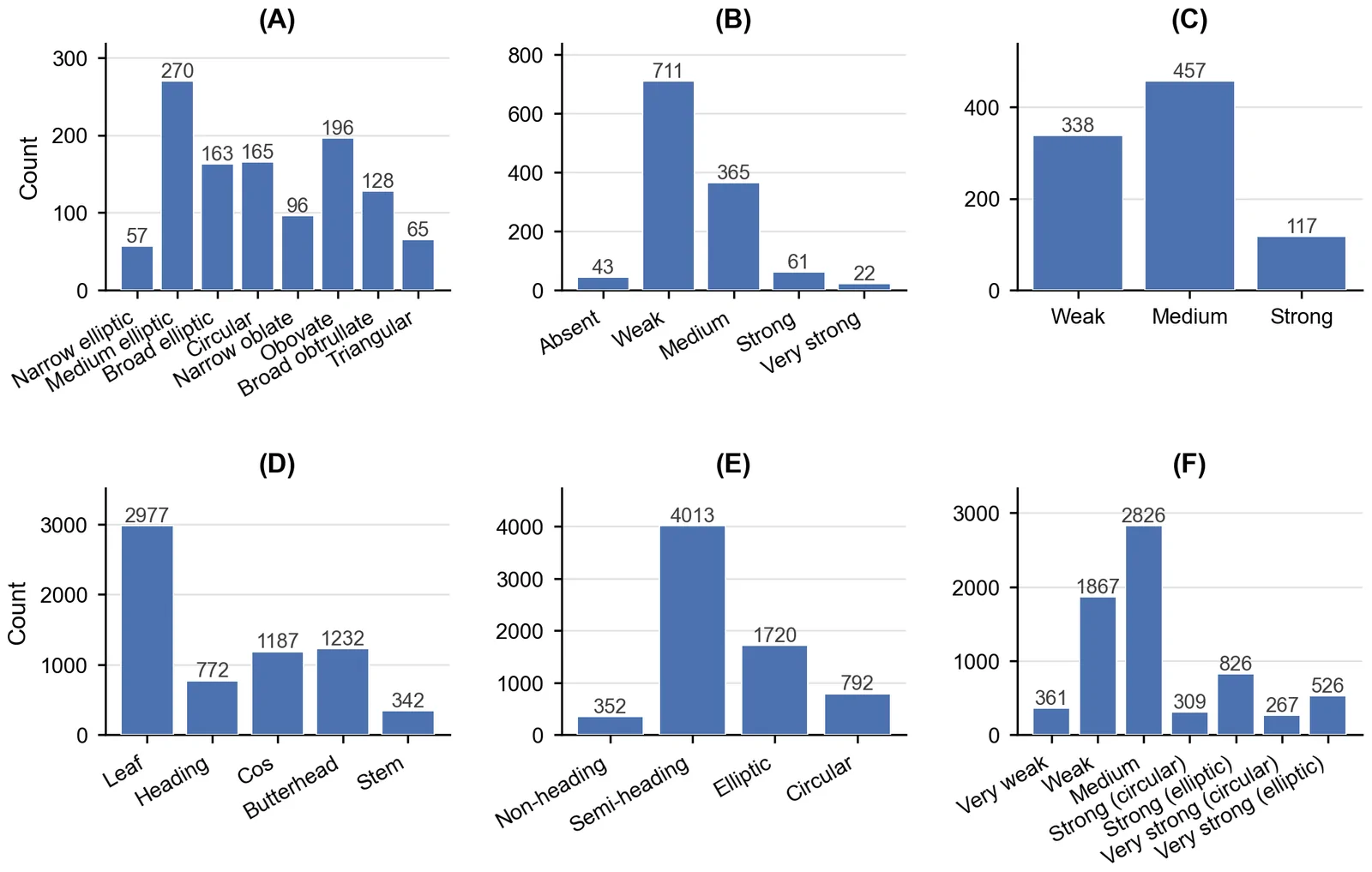
Detailed class distribution for each trait. **(A)** Leaf shape. **(B)** Leaf blistering. **(C)** Leaf margin serration. **(D)** Plant type. **(E)** Head type. **(F)** Heading degree. The value above each bar denotes the number of images in that class.

### 2.2 Basic training conditions

For an objective and fair comparison, all hyperparameters and training conditions other than the training strategy were set identically. To ensure reproducibility, the same set of three random seeds was used for all experiments (Srinivasu et al., 2026), and results are reported as the mean ± standard deviation over the three runs to assess run-to-run variability and stability. The Adam optimizer was used for model optimization, with a learning rate of 1e-4 and a batch size of 16. All models were trained for 50 epochs without early stopping.

Considering the class imbalance inherent in the datasets, a weighted cross-entropy loss was used in which the inverse of the class frequency was assigned as the weight, so that errors on less frequent classes were weighted more heavily during optimization. This loss was applied equally to all training strategies. In addition, random horizontal flipping and random rotation within a range of ±15° were applied to the training data only, so that the models would be less sensitive to variation in the imaging composition and in the position and orientation of the specimen. The ImageNet-1K weights provided by torchvision were used as the pretrained weights, and the input images were normalized with the per-channel means and standard deviations of the ImageNet dataset.

### 2.3 Evaluation metrics

Because performance is difficult to judge from accuracy alone under small-scale and imbalanced conditions, a range of metrics was used together. Specifically, accuracy, precision, recall, specificity, and F1-score were calculated. Macro-averaging was applied to all metrics requiring an average across classes. Each metric is calculated as in Equations 1–5 on the basis of true positives (TP), true negatives (TN), false positives (FP), and false negatives (FN).

(1) $$\text{Accuracy}=\frac{TP+TN}{TP+TN+FP+FN}$$

(2) $$\text{Precision}=\frac{TP}{TP+FP}$$

(3) $$\text{Recall}=\frac{TP}{TP+FN}$$

(4) $$\text{Specificity}=\frac{TN}{TN+FP}$$

(5) $$\text{F}1\text{‐score}=2\times \frac{\text{Precision}\times \text{Recall}}{\text{Precision}+\text{Recall}}$$

### 2.4 Comparative backbones and training strategies

Six convolutional neural networks were used as the comparative backbones in this study: ConvNeXt-Tiny (Liu et al., 2022), DenseNet-121 (Huang et al., 2017), EfficientNet-B0 (Tan and Le, 2019), MobileNetV2 (Sandler et al., 2018), ResNet-18 (He et al., 2016), and VGG16 (Simonyan and Zisserman, 2014). These span a range of capacities and structural characteristics, from standard to lightweight architectures, making them suitable for a comprehensive comparison of the effect of different network structures on learning performance with small-scale data. Each network was configured by changing the final classifier layer to match the number of classes of the trait. Model size, computational cost, and inference speed of each backbone were expressed as the number of parameters, giga floating-point operations (GFLOPs), and frames per second (FPS), respectively, and are given in **Table 2**.

**Table 2:** Parameter count, computational cost, and inference throughput of the comparative backbones.

Backbone	Parameters (M)	GFLOPs	FPS
VGG16	138.36	15.48	742.7
ResNet-18	11.69	1.82	3973.4
DenseNet-121	7.98	2.88	1222.1
EfficientNet-B0	5.29	0.39	2712.7
MobileNetV2	3.50	0.30	3913.3
ConvNeXt-Tiny	28.59	4.46	1061.0

Parameters are given in millions, and GFLOPs per image. Higher FPS indicates faster inference, measured with a batch size of 16 on a single NVIDIA GeForce RTX 3080 Ti.

Three training strategies were used. First, training from scratch is an approach in which no pretrained weights are used and the weights are set according to the default initialization scheme of each architecture and trained from the beginning. As a method that does not make use of learned weights, it can serve as a baseline indicating the level of performance attainable from the genetic resource data alone.

The other two methods are transfer learning techniques that make use of weights pretrained on a large-scale dataset. In the feature extraction approach, the weights of the pretrained backbone network are frozen and only the newly attached classifier is trained. Because few parameters need to be trained, it is known as a low-cost alternative with low training cost and low risk of overfitting (Dobrzycki et al., 2025). Conversely, because the backbone is fixed, gains in performance can be limited if the pretrained features do not sufficiently reflect the characteristics of the target domain (Ghazi et al., 2017).

In the fine-tuning approach, the pretrained weights are taken as initial values and the whole network or some of its layers are retrained together with the newly attached classifier to suit the target dataset. This makes it possible to adapt the representation itself to the target domain, but because more parameters are updated than in feature extraction, the training cost is higher and there is a risk of overfitting depending on the size of the data. In this study, full fine-tuning, in which all layers of the network including the backbone are retrained, was applied.

Crossing the six backbones, three training strategies, and six traits gave a total of 108 experimental conditions. Comparisons between strategies are made in units of the 36 combinations with the same backbone and trait. By comparing the three strategies under identical conditions, the effect of the interaction between network structure and training strategy on genetic resource trait classification performance was analyzed empirically.

To assess statistical differences among the three training strategies, the mean macro F1-score obtained from three independent random seeds was calculated for each backbone–trait combination. The 36 backbone–trait combinations were treated as matched blocks across the three training strategies, and an overall comparison was performed using the Friedman test. When a significant overall difference was detected, *post hoc* pairwise comparisons were conducted using the Wilcoxon signed-rank test with Holm correction for multiple comparisons. Statistical significance was defined as an adjusted *p*-value below 0.05.

## 3 Results

The training performance for the six traits was compared on the basis of the macro F1-score in order to reflect precision and recall in a balanced manner (**Table 3**).

**Table 3:** Macro F1-scores (mean ± SD over three runs) for trait classification by backbone and training strategy. .

Backbone	Strategy	Leaf shape	Leaf blistering	Leaf margin serration	Plant type	Head type	Heading degree
ConvNeXt-Tiny	Training from scratch	0.741 ± 0.041	0.354 ± 0.020	0.662 ± 0.013	0.802 ± 0.010	0.774 ± 0.011	0.695 ± 0.029
	Feature extraction	0.631 ± 0.003	0.357 ± 0.015	0.863 ± 0.010	0.947 ± 0.001	0.919 ± 0.005	0.805 ± 0.004
	Fine-tuning	0.823 ± 0.013	0.558 ± 0.109	0.903 ± 0.018	0.985 ± 0.008	0.975 ± 0.008	**0.899 ± 0.008**
DenseNet-121	Training from scratch	0.734 ± 0.014	0.579 ± 0.044	0.814 ± 0.024	0.936 ± 0.007	0.918 ± 0.014	0.816 ± 0.005
	Feature extraction	0.642 ± 0.032	0.476 ± 0.045	0.824 ± 0.016	0.935 ± 0.002	0.881 ± 0.010	0.759 ± 0.003
	Fine-tuning	**0.828 ± 0.009**	0.613 ± 0.011	0.892 ± 0.034	0.980 ± 0.005	0.972 ± 0.005	0.880 ± 0.014
EfficientNet-B0	Training from scratch	0.766 ± 0.022	0.464 ± 0.058	0.754 ± 0.069	0.898 ± 0.015	0.875 ± 0.026	0.773 ± 0.017
	Feature extraction	0.612 ± 0.019	0.447 ± 0.024	0.784 ± 0.016	0.915 ± 0.018	0.871 ± 0.005	0.718 ± 0.009
	Fine-tuning	0.810 ± 0.044	0.621 ± 0.026	0.904 ± 0.013	**0.988 ± 0.004**	**0.978 ± 0.002**	0.889 ± 0.002
MobileNetV2	Training from scratch	0.734 ± 0.008	0.496 ± 0.033	0.735 ± 0.023	0.877 ± 0.019	0.839 ± 0.022	0.746 ± 0.019
	Feature extraction	0.623 ± 0.035	0.497 ± 0.026	0.874 ± 0.022	0.939 ± 0.008	0.881 ± 0.003	0.765 ± 0.010
	Fine-tuning	0.813 ± 0.001	**0.642 ± 0.053**	**0.908 ± 0.026**	0.980 ± 0.004	0.969 ± 0.007	0.866 ± 0.019
ResNet-18	Training from scratch	0.766 ± 0.022	0.481 ± 0.032	0.791 ± 0.012	0.899 ± 0.025	0.871 ± 0.045	0.748 ± 0.061
	Feature extraction	0.540 ± 0.023	0.417 ± 0.029	0.756 ± 0.018	0.903 ± 0.002	0.868 ± 0.010	0.728 ± 0.015
	Fine-tuning	0.784 ± 0.019	0.621 ± 0.059	0.902 ± 0.017	0.946 ± 0.044	0.966 ± 0.005	0.855 ± 0.003
VGG16	Training from scratch	0.796 ± 0.010	0.342 ± 0.167	0.813 ± 0.052	0.599 ± 0.411	0.857 ± 0.020	0.471 ± 0.337
	Feature extraction	0.619 ± 0.016	0.428 ± 0.007	0.802 ± 0.038	0.902 ± 0.003	0.822 ± 0.013	0.683 ± 0.016
	Fine-tuning	0.792 ± 0.012	0.475 ± 0.114	0.859 ± 0.023	0.948 ± 0.028	0.933 ± 0.014	0.819 ± 0.034

The highest mean value for each trait is shown in bold.

Based on the mean over three runs, fine-tuning recorded the highest macro F1-score in 35 of the 36 backbone–trait combinations. The exception was leaf shape with VGG16, for which training from scratch reached 0.796 ± 0.010, slightly exceeding the 0.792 ± 0.012 of fine-tuning, with a difference smaller than the run-to-run standard deviation. In addition, the mean for fine-tuning was the highest for all six traits (**Figure 4**), and the values for the individual backbones were also for the most part distributed at their highest positions under fine-tuning. Across the 36 paired conditions, the mean macro F1-score was 0.849 for fine-tuning, 0.728 for training from scratch, and 0.734 for feature extraction. The difference among the three strategies was significant (Friedman *χ*^2^ = 51.17, p< 0.001), and *post-hoc* tests showed that fine-tuning significantly outperformed both training from scratch and feature extraction (adjusted p< 0.001 for both), whereas the latter two did not differ significantly (adjusted p = 0.932).

**Figure 4:**
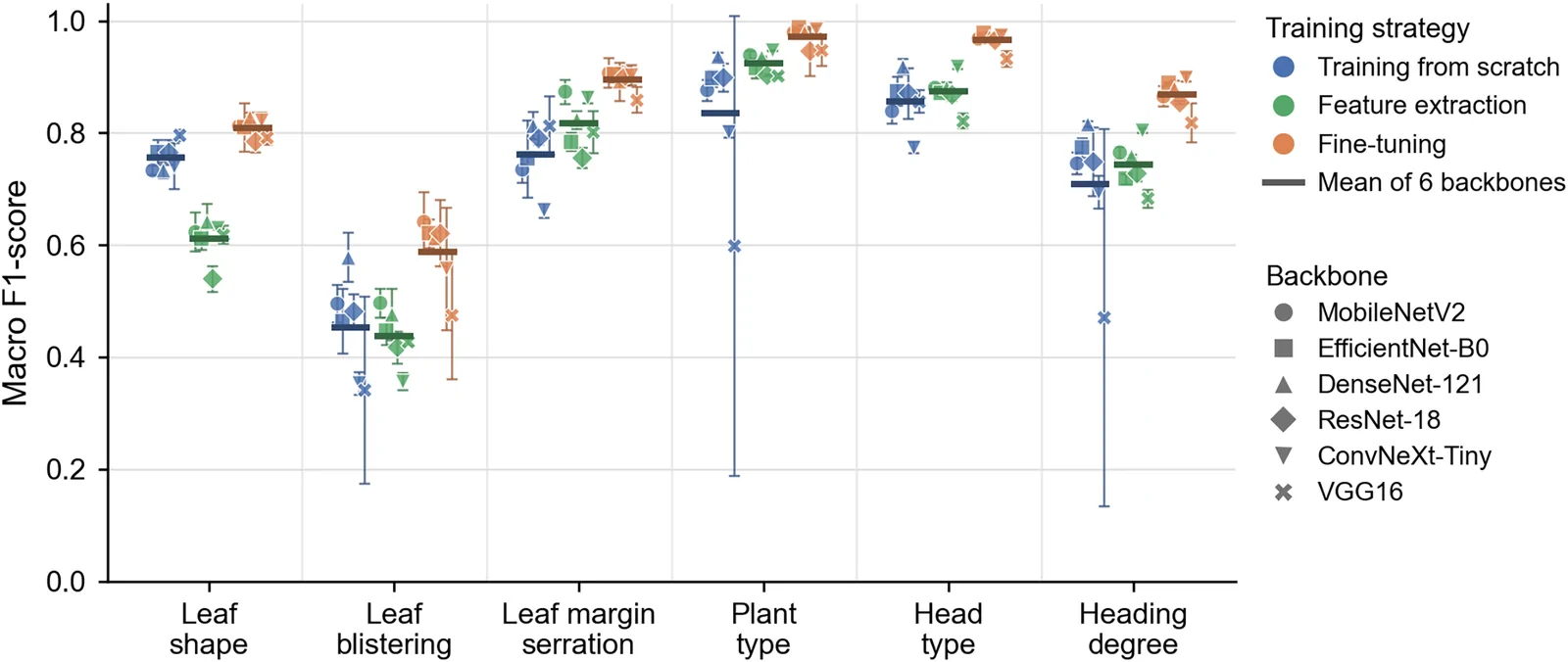
Macro F1-scores by trait and training strategy. Each marker represents the mean macro F1-score from three independent runs with different random seeds for one backbone, and vertical error bars indicate ±1 standard deviation across the three runs. The horizontal bar represents the mean across the six backbones for each training strategy.

However, such consistency was not observed among the backbones. The backbone with the highest mean performance varied by trait: EfficientNet-B0 performed best for plant type and head type, MobileNetV2 for leaf blistering and leaf margin serration, DenseNet-121 for leaf shape, and ConvNeXt-Tiny for heading degree. Thus, no single backbone was consistently superior across all traits.

Although the performance advantage of fine-tuning was consistent across most conditions, the level of performance attained differed among traits. Leaf blistering, photographed as single leaves, had the lowest value of the six traits, with a best combination of 0.642 ± 0.053 (MobileNetV2 with fine-tuning), whereas for plant type and head type, photographed as whole plants, all combinations except training from scratch with VGG16 reached 0.77 or above. That exception, the combination of plant type with VGG16 under training from scratch, showed a low mean with particularly large run-to-run variability (0.599 ± 0.411), indicating high sensitivity to stochastic training conditions rather than consistently poor performance. Relatively large variability was also observed for VGG16 trained from scratch on heading degree (0.471 ± 0.337) and leaf blistering (0.342 ± 0.167).

The three strategies were compared across five metrics within the best backbone for each trait (**Figure 5**). Fine-tuning recorded the highest values for all six traits. The extent to which differences between strategies were apparent varied with the metric, however. The values of the three strategies were close to one another for specificity, whereas the differences were clear for accuracy, precision, recall, and F1-score. In the multi-class setting, all classes but one are treated as negative, so negative samples account for a large proportion and specificity consequently remains high. For plant type and head type, the performance of training from scratch and feature extraction was almost identical, whereas for leaf shape training from scratch gave better results than feature extraction. For some traits, meanwhile, accuracy was higher than the macro F1-score, and the difference was most pronounced for leaf blistering. Predictions biased toward the majority classes raise overall accuracy while the macro F1-score, which reflects the minority classes equally, remains low, so the difference between the two metrics indicates a state in which performance on the minority classes has not been secured.

**Figure 5:**
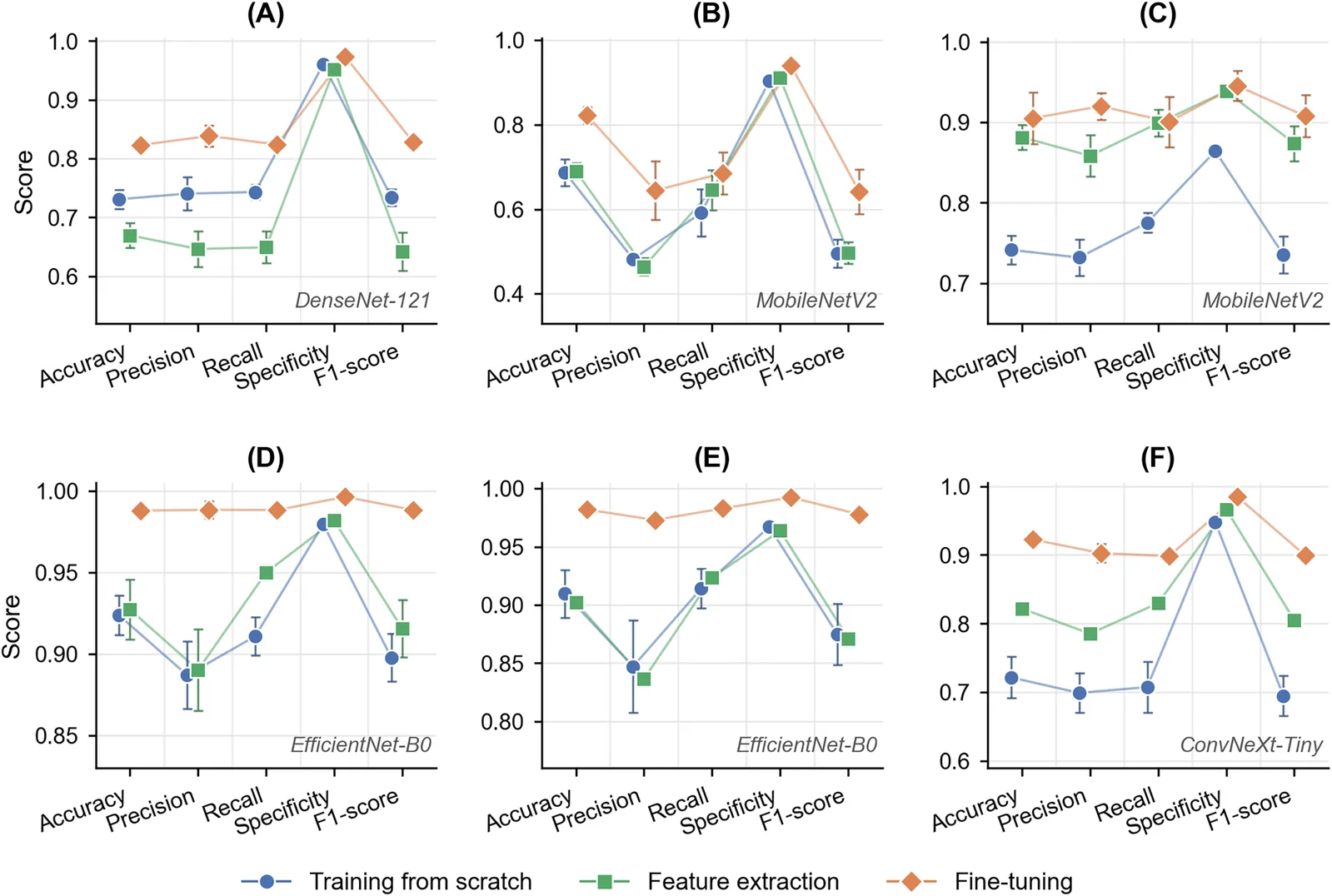
Comparison of five classification metrics among the three training strategies for the selected backbone of each trait. For each trait, the backbone corresponding to the highest mean macro F1-score among all backbone–strategy combinations was selected based on three independent runs. Markers represent the mean across the three runs, and vertical error bars indicate ±1 standard deviation. **(A)** Leaf shape. **(B)** Leaf blistering. **(C)** Leaf margin serration. **(D)** Plant type. **(E)** Head type. **(F)** Heading degree.

The pattern of misclassification was examined in the confusion matrices (**Figure 6**). Plant type and head type showed recall of 0.95 or above for most classes and were close to diagonal in form, whereas leaf shape, which has many classes, and leaf blistering, which has severe class imbalance, showed more misclassification even for their best combinations. Some classes of leaf blistering have very few samples, however, so interpretation requires caution.

**Figure 6:**
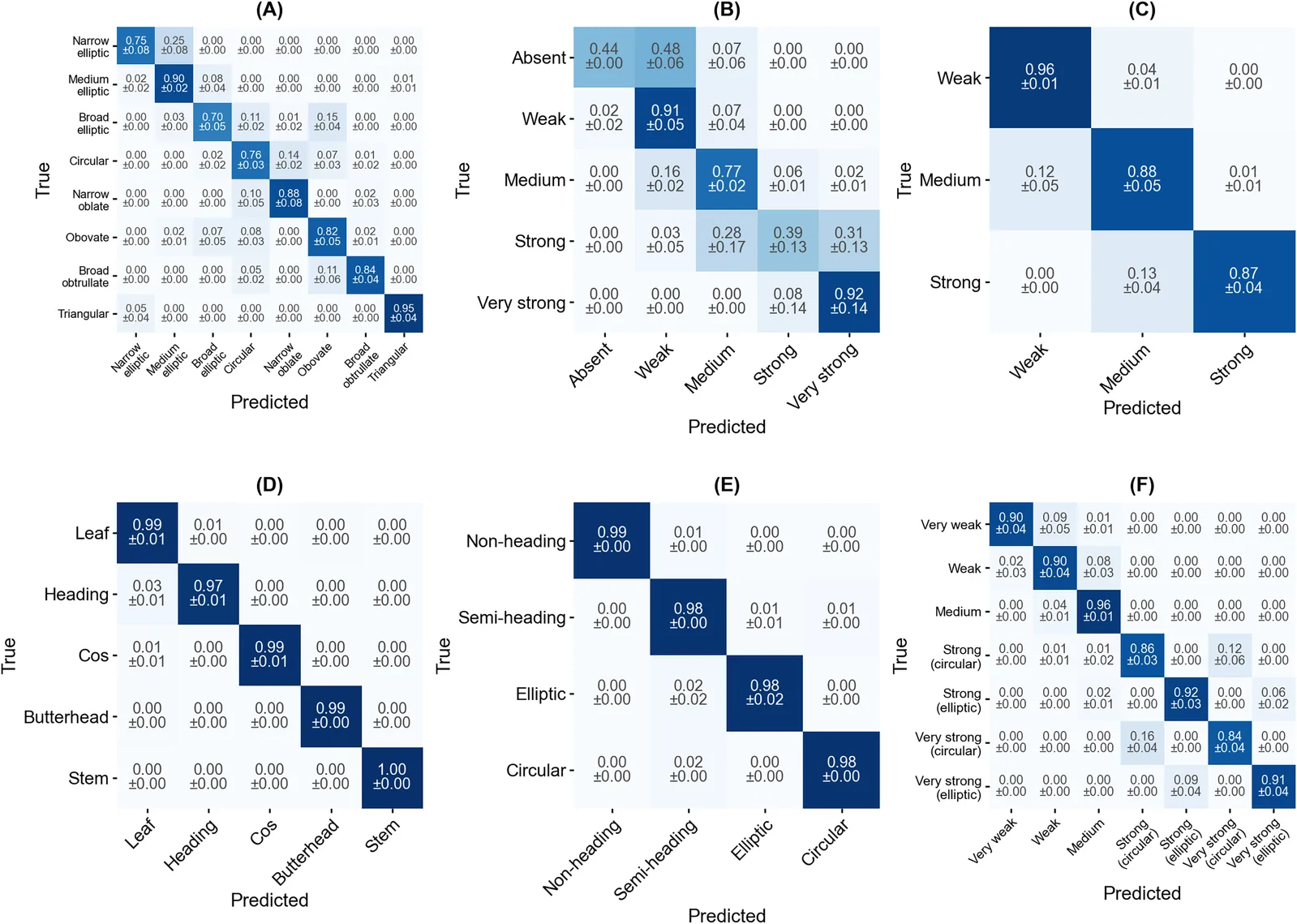
Confusion matrices for the backbone–strategy combination achieving the highest mean macro F1-score across three independent runs for each trait. For each run, the values were normalized by the number of true samples in each class, such that each row sums to approximately 1. Each cell reports the mean normalized value across the three runs, with ±1 standard deviation. **(A)** Leaf shape: DenseNet-121 with fine-tuning. **(B)** Leaf blistering: MobileNetV2 with fine-tuning. **(C)** Leaf margin serration: MobileNetV2 with fine-tuning. **(D)** Plant type: EfficientNet-B0 with fine-tuning. **(E)** Head type: EfficientNet-B0 with fine-tuning. **(F)** Heading degree: ConvNeXt-Tiny with fine-tuning.

Misclassifications were also concentrated in morphologically adjacent classes rather than being randomly distributed. For leaf shape, confusion between neighboring classes appeared in the narrow elliptic, medium elliptic, and broad elliptic series, in which the width changes gradually, and bidirectional confusion was also observed among broad elliptic, obovate, and circular, which share a rounded outline. For leaf blistering as well, confusion between adjacent classes occurred chiefly in the form of strong being absorbed into medium and absent into weak, while confusion skipping a class was rare. Heading degree has a structure in which some classes are subdivided into circular and elliptic, and there was almost no confusion in this division between subclasses, with misclassification concentrated between adjacent intensity classes.

For the traits with an ordinal structure (leaf blistering, leaf margin serration, and heading degree), additional metrics reflecting the distance between classes were calculated (**Table 4**), with each class represented by its index on the ordinal scale. MAE is the mean of the absolute difference between the predicted and true class indices, and Acc@1 is the proportion of predictions whose class index differs from the true one by at most one. QWK (Cohen, 1968) compares the observed confusion matrix with the one expected from the marginal class distributions, weighting each error by the squared distance between classes, so that agreement arising by chance is discounted. All three treat the classes of a trait as equally spaced.

**Table 4:** Class-distance-based evaluation metrics for the ordinal traits by training strategy.

Trait	Strategy	Macro F1	QWK	MAE	Acc@1
Leaf blistering	Training from scratch	0.452 ± 0.022	0.606 ± 0.052	0.383 ± 0.030	0.962 ± 0.001
	Feature extraction	0.437 ± 0.017	0.607 ± 0.009	0.436 ± 0.001	0.939 ± 0.002
	Fine-tuning	**0.588 ± 0.022**	**0.782 ± 0.007**	**0.208 ± 0.021**	**0.987 ± 0.003**
Leaf margin serration	Training from scratch	0.762 ± 0.020	0.750 ± 0.021	0.224 ± 0.022	0.995 ± 0.003
	Feature extraction	0.817 ± 0.018	0.826 ± 0.012	0.169 ± 0.014	**1.000 ± 0.001**
	Fine-tuning	**0.895 ± 0.009**	**0.900 ± 0.008**	**0.085 ± 0.007**	**1.000 ± 0.000**
Heading degree	Training from scratch	0.708 ± 0.069	0.790 ± 0.083	0.296 ± 0.053	0.956 ± 0.013
	Feature extraction	0.743 ± 0.003	0.846 ± 0.003	0.280 ± 0.005	0.963 ± 0.002
	Fine-tuning	**0.868 ± 0.011**	**0.944 ± 0.006**	**0.111 ± 0.011**	**0.994 ± 0.002**

Each value is the mean ± SD over three random seeds, where the value for each seed is the mean over the six backbones, and the best mean value for each trait and metric is shown in bold. For heading degree, the macro F1-score is based on seven classes and the remaining metrics on the five-level intensity scale described in the text.

For leaf blistering and leaf margin serration, the order of the classes corresponds directly to the intensity level, so the distance between classes was used as it is. For heading degree, strong and very strong among the seven classes are each subdivided into circular and elliptic, so the same intensity level was assigned to the two subclasses, converting the scale to five levels before the class distances were calculated.

According to **Table 4**, predictions deviating by two or more classes were not frequent even for the traits whose macro F1-scores were relatively low. The mean Acc@1 values were 0.939 or above across all nine combinations, and 0.987 or above for all three traits under fine-tuning. The MAE under fine-tuning was likewise 0.208 or less, below one class, and the QWK was 0.782 or above. This supports the view that even where predictions did not match the true class exactly, the difference generally remained within one class.

The regions to which the models attended when making predictions were also visualized using Grad-CAM (Selvaraju et al., 2017). Grad-CAM is a technique that displays the regions contributing to the decision as a heatmap by taking a weighted sum of the gradients of the predicted class score over the feature maps of the last convolutional layer. For each trait, one image was randomly selected from the test set, and the same input image was used to compare the six backbones (**Figure 7**). In these examples, for most of the backbones, activation formed within the plant or leaf region rather than the background, and even in images of plants that also included surrounding specimens and background, activation was concentrated on the central specimen. This suggests that regions of the target specimen contributed to the predictions in these cases. The location and extent of activation differed between backbones, however, and in some cases of misclassification activation was also observed to be dispersed across several locations.

**Figure 7:**
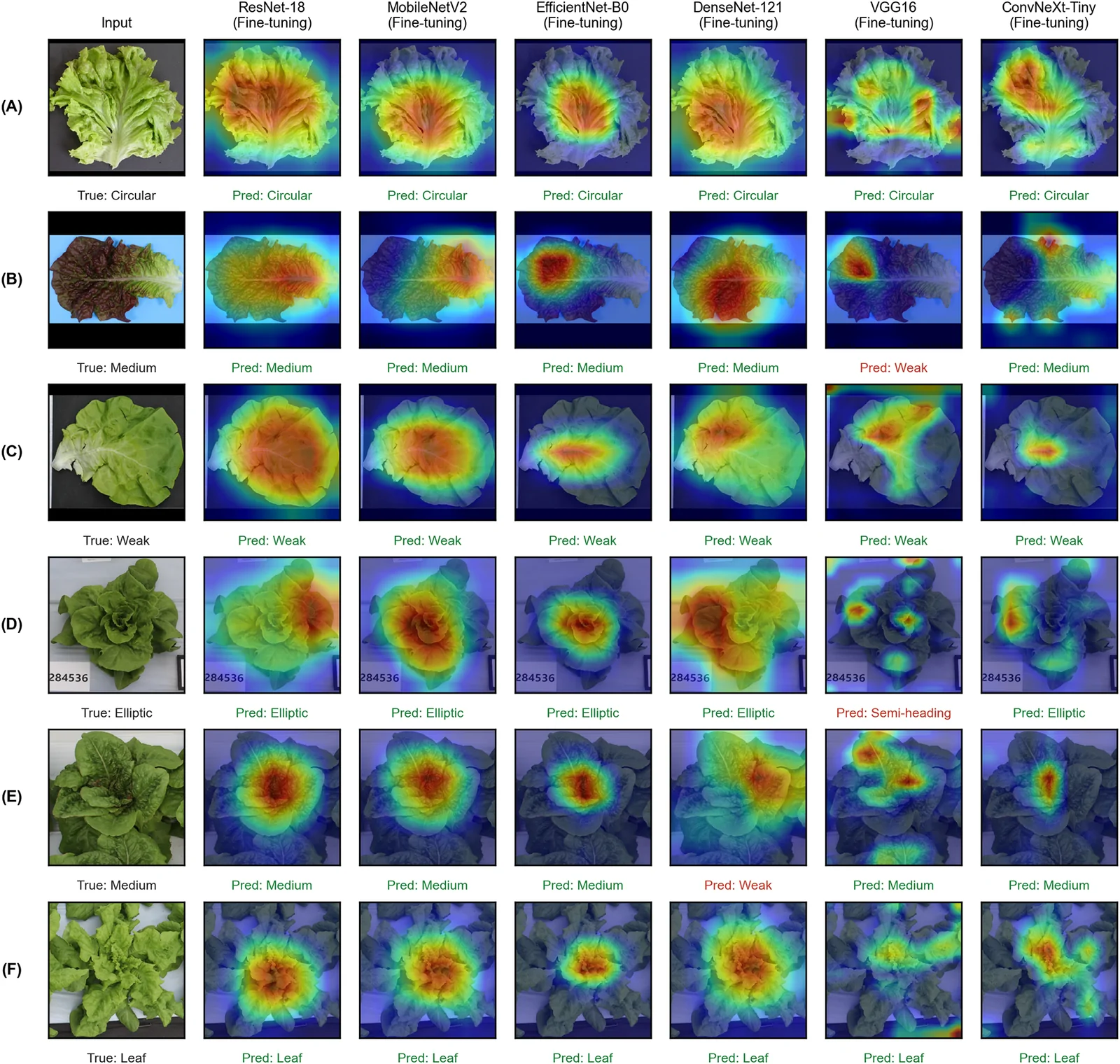
Grad-CAM heatmaps generated by the fine-tuned models of the six backbones for one test image randomly selected for each trait. **(A)** Leaf shape. **(B)** Leaf margin serration. **(C)** Leaf blistering. **(D)** Head type. **(E)** Heading degree. **(F)** Plant type. The leftmost column shows the input image and the remaining columns the corresponding heatmaps. The label below each image denotes the predicted class, with green indicating a correct prediction and red a misclassification.

## 4 Discussion

In this study, fine-tuning showed the highest performance in most combinations, whereas the relative ranking of training from scratch and feature extraction varied by trait. The two transfer learning strategies use the same pretrained weights but differ in whether the backbone is allowed to update, so the usefulness of the frozen representation may have depended on the degree of overlap between the pretrained representation and the visual information required by the trait. This performance gap appeared relatively large for traits that have many classes and depend on subtle differences in outline, which partly illustrates the point at which images of agricultural genetic resources differ from images of general objects. The design of this study was oriented toward comparing performance between strategies, however, and did not verify this explanation directly.

With respect to the backbones, no particular architecture was superior across all traits, and the variation in performance between backbones also differed by trait. This difference was clearly observed with the VGG16 model. This backbone not only showed large run-to-run variability under training from scratch but also showed activation dispersed across several locations in the Grad-CAM examples. This suggests that the suitability of a backbone for genetic resource data may differ depending on its structural characteristics. When designing a classification model, it is therefore necessary to consider both the visual characteristics of the target data and the suitability of the backbone. The Grad-CAM analysis in this study was a qualitative examination limited to a small number of cases per trait, however, so quantitative evaluation will need to accompany it in future in order to generalize the relationship between the regions to which a model attends and its classification performance.

Model performance also did not increase with computational complexity. Under fine-tuning, EfficientNet-B0 and MobileNetV2 achieved mean performance comparable to or higher than that of larger networks, whereas VGG16, which had the largest parameter count and computational cost, showed the lowest mean performance (**Tables 3** and **2**). Measured inference throughput also differed among backbones and did not correspond directly to parameter count or GFLOPs. These results suggest that practical model selection should consider predictive performance together with computational cost and measured inference efficiency.

The finding that the upper limit of performance attainable through different training strategies varied among traits appears to reflect characteristics inherent in the dataset. For traits with severe class imbalance, performance on minority classes remained limited even under the best-performing strategy, whereas traits with continuous class boundaries showed persistent misclassification between adjacent classes. The rarity of errors spanning two or more classes on the ordinal metrics suggests that this limitation may arise more from ambiguity at adjacent-class boundaries than from a general failure of trait discrimination. By contrast, for traits such as plant type and head type, all three strategies achieved relatively high performance, leaving less room for improvement than for traits such as leaf blistering. Thus, although optimization of the transfer learning strategy can improve performance, the magnitude of the gain is constrained in part by the sample distribution and class definitions of each trait. Continuous class boundaries may also introduce label uncertainty, meaning that some adjacent-class errors may reflect annotation uncertainty as well as model limitations. Because inter- and intra-observer agreement was not quantified in this study, these contributions could not be disentangled. Future studies could better characterize and reduce such adjacent-class errors by quantifying annotation uncertainty and incorporating ordinal-aware learning objectives that account for class order.

These results suggest that the scope of application of an automated system needs to be set differently according to the phenotype of the lettuce genetic resource. For traits that showed relatively high performance across strategies, the classification model could support routine screening or the prioritization of expert review, while for traits whose performance was limited, expert assessment should remain the primary basis of determination. For traits whose performance plateaus even when the strategy is optimized, meanwhile, the inherent imbalance of the dataset may be one contributing factor, although this study did not isolate its effect from that of the model. Given the nature of agricultural genetic resources in particular, accessions with a specific phenotype are themselves rare, so there are practical difficulties in correcting the distribution by physically expanding data collection. Applying automated classification to such traits therefore requires that factors other than the training strategy be considered as well. In this sense, the models are better positioned as decision-support tools than as replacements for expert evaluation, and direct comparison with inter- or intra-rater agreement will be required before any reduction in evaluator variability can be claimed.

The images in this study were accumulated over 14 years with different imaging equipment and lighting conditions, so resolution, background, and imaging distance are not uniform. This heterogeneity is a factor that raises the difficulty of training compared with data acquired under controlled experimental conditions, but it at the same time reflects the characteristics of the archival material that genebanks have accumulated over long periods. The results of this study therefore provide initial evidence on the potential use of existing accumulated material without newly controlling the imaging environment.

The present study, however, evaluated phenotype classification using a random split at the individual-sample level rather than generalization to previously unseen accessions. Because the split was not grouped by accession, different individuals originating from the same accession could occur in different subsets, and the reported performance should therefore be interpreted within the scope of individual-level phenotype classification. Likewise, the random split was not designed to evaluate generalization to unseen acquisition years, cameras, or imaging sessions. Accession-level and acquisition-domain holdout validation, where metadata permit, will be necessary to assess generalization to new genetic resources and imaging conditions, and represents an important direction for future work.

Finally, this study was limited to six traits of lettuce, so additional validation is required to generalize the findings to other crops or traits. In addition, because the same hyperparameters were applied to ensure comparable conditions across strategies, the best performance achievable under conditions individually optimized for each strategy could not be evaluated. Similarly, weighted cross-entropy was applied as a common condition, and its effect was not directly compared with standard cross-entropy or other imbalance-aware approaches. Future studies that examine these factors under a broader range of experimental conditions could provide a more precise assessment of model performance and applicability in genebank settings.

## 5 Conclusion

To identify a training strategy suited to image-based support of trait characterization in genebanks, this study crossed six convolutional neural networks with three training strategies for six morphological traits of lettuce genetic resources, trained them under identical conditions with three different random seeds, and compared their performance. Fine-tuning recorded the highest mean macro F1-score in 35 of the 36 backbone–trait combinations and significantly outperformed training from scratch and feature extraction. For small-scale and imbalanced images of genetic resources, pretrained weights were effective when used as initial values rather than as a fixed feature extractor. No single backbone was superior across all traits, by contrast, and backbones with smaller parameter counts recorded the best performance more frequently.

The performance attainable through the choice of strategy differed among traits. The analysis of misclassification showed that the plateau in performance may be related to the discrimination of morphologically adjacent classes. Errors were not randomly distributed but were concentrated in adjacent classes in which the form changes continuously, and on the metrics reflecting class distance, misclassifications deviating by two or more classes were likewise infrequent. This suggests that the continuous boundaries between the classes of these traits, which give adjacent classes a high degree of morphological similarity, and possibly uncertainty in the labeling of neighboring categories contribute to the remaining classification difficulty.

It was also possible to distinguish traits for which the training strategy alone achieved relatively high performance from those for which it did not. The results of this study can serve as empirical evidence for genebanks in determining the role of model-assisted characterization on a trait-by-trait basis. For traits whose performance has plateaued, however, additional elements such as training methods that reflect the class structure and the uncertainty of labels need to be introduced in addition to the choice of backbone and training strategy.

## Data Availability

The raw data supporting the conclusions of this article will be made available by the authors, without undue reservation.
