# Supplementary material for: Fine-tuning improves deep learning-based digital phenotyping of lettuce genetic resources
Source: Front Plant Sci. 2026 Oct 9;17:1970840. doi: 10.3389/fpls.2026.1970840 (PMC13690775; doi:10.3389/fpls.2026.1970840)
Supplement: Supplementary file 1 [file DataSheet1.pdf]

Supplementary Material

1 SUPPLEMENTARY TABLES

Table S1. Class distribution of the training, validation, and test sets for each trait.

Trait	Class	Train	Validation	Test	Total
Leaf shape	Narrow elliptic	34	11	12	57
	Medium elliptic	162	54	54	270
	Broad elliptic	98	33	32	163
	Circular	99	33	33	165
	Narrow oblate	57	19	20	96
	Obovate	118	39	39	196
	Broad obtrullate	77	26	25	128
	Triangular	39	13	13	65
Leaf blistering	Absent	25	9	9	43
	Weak	426	142	143	711
	Medium	219	73	73	365
	Strong	37	12	12	61
	Very strong	13	5	4	22
Leaf margin serration	Weak	203	67	68	338
	Medium	274	91	92	457
	Strong	70	24	23	117
Plant type	Leaf type	1787	595	595	2977
	Heading type	463	154	155	772
	Cos type	712	238	237	1187
	Butterhead type	739	246	247	1232
	Stem type	205	69	68	342
Head type	Non-heading	211	71	70	352
	Semi-heading	2408	802	803	4013
	Elliptic	1032	344	344	1720
	Circular	475	158	159	792
Heading degree	Very weak	217	72	72	361
	Weak	1120	373	374	1867
	Medium	1695	565	566	2826
	Strong (circular)	185	62	62	309
	Strong (elliptic)	496	165	165	826
	Very strong (circular)	160	54	53	267
	Very strong (elliptic)	316	105	105	526
